# Creativity development of tourism villages in Bandung Regency, Indonesia: co-creating sustainability and urban resilience

**DOI:** 10.1038/s41598-023-49094-1

**Published:** 2024-01-16

**Authors:** Rd Ahmad Buchari, Abdillah Abdillah, Ida Widianingsih, Heru Nurasa

**Affiliations:** 1 Department of Public Administration, Faculty of Social and Political Sciences, Padjadjaran University, Bandung, Indonesia; 2 Center for Decentralization and Participatory Development Research, Faculty of Social and Political Sciences, Padjadjaran University, Bandung, Indonesia; 3 Graduate Program in Administrative Science, Faculty of Social and Political Sciences, Padjadjaran University, Bandung, Indonesia

**Keywords:** Environmental sciences, Environmental social sciences, Environmental economics, Socioeconomic scenarios, Sustainability

## Abstract

This study aims to explore the interactions between the government, the tourism industry, universities, media, society, and the environment in the management and utilization of tourist villages in Bandung Regency, Indonesia. The research employed a qualitative-explorative method with a case study approach. Research analysis was assisted by the Nvivo 12 Plus qualitative analysis tool. The result's findings show that tourist villages require coordinated efforts from the government, tourism sector, universities, media, local communities, and the environment because in our opinion the environment/nature cannot only be viewed as an object but must rather be aligned with other important sectors in development programs. This connection may at the very least provide a means of enhancing the management of tourism villages and achieving sustainability and resilience. The contribution of this research provides insight into the process of developing creative tourism villages in realizing sustainability and resilience through tourism villages that pay attention to economic, social, infrastructure, and environmental dimensions.

## Introduction

Currently, Indonesia is encouraging development through the development of tourist villages as an alternative choice to maintain sustainable economic development in remote/rural areas with involving various actors/sectors^1,2^. Interaction with other stakeholders, such as government, private, and community, is necessary to maintain sustainable economic development in rural areas in good governance^3,4^. The combination of these three factors—government, business, and society—does not, however, suffice to promote the development of tourism in light of the changing times^5,6^. Therefore, the role of other actors/sectors such as the environment, media, and higher education institutions is very important in supporting tourism development^7^. According to Hall and Williams^8^, it is crucial to recognize innovation as a crucial component of the entire tourism system given the changes in the business. Each development sector has an important role in promoting sustainability. Such as higher education can produce knowledge of innovation, the environment has resources that can be utilized in innovation, the media encourages participation in development, the industry provides economic resources and market potential to absorb innovation, and the government sets standards and innovation of incentive policies^7,9,10^.

Bandung Regency, Indonesia is one of the areas with the creative development of tourist villages in Indonesia, this is due to the Bandung Regency government program which launched the development of 100 tourist villages^11,12^. This program is recognized as an effort to exploit natural and cultural potentials, empower communities, and accelerate economic growth. According to a report from the Head of the Bandung Regency Government Disparbud, as many as 50 of the 100 targeted tourism villages have now been designated as pilot villages^11,12^. Until 2022, ten (10) villages have been designated as tourist villages through the Regent's Decree^11–13^.

Referring to the Decree of the Regent of Bandung Regency Number 556.42/Kop7-Dispopar/2011, the ten tourism villages in question are Alam Endah Village, Gambung Village, Penundaan Village, Lebak Muncang Village, Lamajang Village, Jelekong, Ciburial Village, Cibolerang Village, Laksana Village, and Rawabogo Village^11–13^. The development of tourist villages can stimulate the economic growth of the surrounding community. Apart from that, there are problems that arise such as a lack of adequate infrastructure and accessibility. Many tourist villages are still difficult to reach by public transportation and lack basic facilities such as good roads, electricity, clean water and adequate sanitation which is a concern in the creative development of tourist villages in Bandung Regency, Indonesia^11–14^. According to Ying and Zhou^1^ and Qin et al.^2^ The problems of tourism village development can only be resolved through interaction between the right actors/sectors so that village sustainability and independence can be realized. In a news tracking report by Ripaldi^12^. it is stated that the Bandung Regency Government is seeking villages that do not have natural potential, to be developed into artificial tourism villages while continuing to encourage local cultural wisdom that exists in the area. Like culinary, cultural arts, and others, it is a part that needs to be developed^11,12^. Recognizing the importance of the foundation of sustainable tourism in every development of the tourist village. That's why the Bandung Regency Government changed the way village development was carried out. Not top-down, but from community participation or desire. If the community's desire is getting higher and stronger, it will be encouraged and carried out well together^11,12^. This is the empirical issue behind which this research was built to explain how actors/sectors interact in the creative development that is being pursued in Bandung Regency, Indonesia. This is explored through the conceptual approach of the Quintuple-Helix innovation model^9^.

A helix element is introducing the environment principle from Quintuple-Helix concepts to promote sustained inventive growth^7,9,10^. Carayannis et al.^9^ discuss the Quintuple-Helix innovation model as a framework for knowledge, innovation, and long-term competitive advantage. Quintuple-helix be considered as a driver of sustainable competitiveness and prosperity in an inventive development by including a fifth element, namely the natural environment^7,9,10^.

In addition, the media, along with the government, the private sector, higher education institutions, and the community, is a key component of the Penta-helix model and plays a significant role in the creative development that promotes the expansion of tourist villages^14–16^. Ayalew^14^ asserts that the media can aid in promoting tourism potential. Pentahelix is an addition to the triple-helix method that involves aspects of organizations and non-profit organizations to realize innovation, according to Muhyi et al.^17^. He continued by saying that the media is a stakeholder that is crucial for promoting and informing business, particularly company development. Additionally, the media is important in promoting local tourism, according to Sumarto et al.^18^. In order to improve tourist management, there must be interactions between the government, the tourism sector, academic institutions, local communities, the media, and the environment. To manage tourism villages more effectively, synergistic relationships between the six helixes should be developed^17,18^. The hilex concept has become a trend in regional development which is used by regional governments in Indonesia to encourage participatory and inclusive development by involving various actors/stakeholders^14–17^.

The research problem statement is how the government, the tourism industry, society, higher education institutions, the media, and the environment interact in tourism management and development of tourist villages in Bandung Regency, Indonesia, based on the background that has been explained. Based on the problem formulation, the researcher tried to study it in depth and comprehensively using a qualitative-explorative methodology approach, this is because this method chooses the advantage of exploring and interpreting critically and in depth the research cases studied which cannot be explained with numbers.

Various studies show that the management of tourism villages requires synergistic interaction of several elements which include the government, the tourism industry, higher education institutions, the media, the community, and the environment^17–20^. At the very least, interaction can help to improve the management of tourist villages. Triple-Helix focuses on conversations on innovation between higher education institutions, businesses, and the government. Media-based culture and the public are added as a fourth helix in the quadruple-helix model. The quintuple-helix combines the components of the quadruple-helix with the social environment's natural surroundings, Penta-Helix has media components. The management of tourism villages in Bandung Regency improves and draws more visitors thanks to the government, the tourism sector, higher education institutions, the media, the community, and the environment that interact with one another. Finally, preserve sustainable economic development in rural areas while promoting the economic growth of the neighborhood. In managing a tourism village in Bandung Regency, Indonesia, this study focuses on the connections between the government, the tourism sector, higher education institutions, media, society, and the environment. This paper includes an introduction, methods, results and discussion, conclusions, and recommendations (the range of further research). Based on various previous studies, development principles in Indonesia tend to see the environment as an object of development and the penta helix concept is more often used in planning development programs and making development policies^14–20^, so the position of this research tries to explore the creative development being pursued in Bandung Regency by looking at the environment as a subject of development that is parallel to other helixes.

### Objective

This study aims to explore the interactions between the government, the tourism industry, universities, media, society, and the environment in the management and utilization of tourist villages in Bandung Regency, Indonesia. This study examines how interaction between stakeholders can encourage the creative development of tourist villages so that they can create sustainability and resilience in Bandung Regency, Indonesia.

## Methods

### Research design (data collections and analysis techniques)

The method used is qualitative-explorative through a case study approach, which highlights the case of the creative development of a tourist village in Bandung Regency, Indonesia. Researchers act as key instruments and researchers explain the complex nature of the problems studied^21^. Determine connected components by merging different viewpoints so that they can be explained holistically from different angles of the processes and events under study. The qualitative-exploratory method was chosen with the aim of exploring and revealing critically and in-depth the research case being studied. Primary data were obtained for this study using observational data (1. Field Notes; 2. Webinars; and 3. Research Forum Discussions) using collection techniques that included open-ended, unstructured general inquiries. Meanwhile, secondary data was collected through document studies, searching Bandung Regency government websites, Indonesia, and online media, surveying various relevant policies, and studying various literature from several related articles. According to Miles et al.^22^, the collected data was processed by compressing, presenting, and drawing conclusions so that the data could be properly accommodated without any loss in findings. The qualitative analysis methods used in this study span the gamut from confirmation to investigation and involve the analysis of textual, visual, or auditory data. Whereas, according to Mihas^23^, qualitative studies can be influenced more by the data itself or more by a conceptual framework, indicating an inductive process. As opposed to attempting to situate their work within a specific epistemological or ontological framework, generic or basic qualitative research refers to methods where researchers are merely interested in finding solutions, bringing about change, or uncovering pertinent themes. This study employs Nvivo 12 Plus tools as qualitative analysis software to explore research data and draw the best conclusions while reducing bias in qualitative analysis^24^.

### Data analysis using Nvivo 12 plus

**Figure 1:**
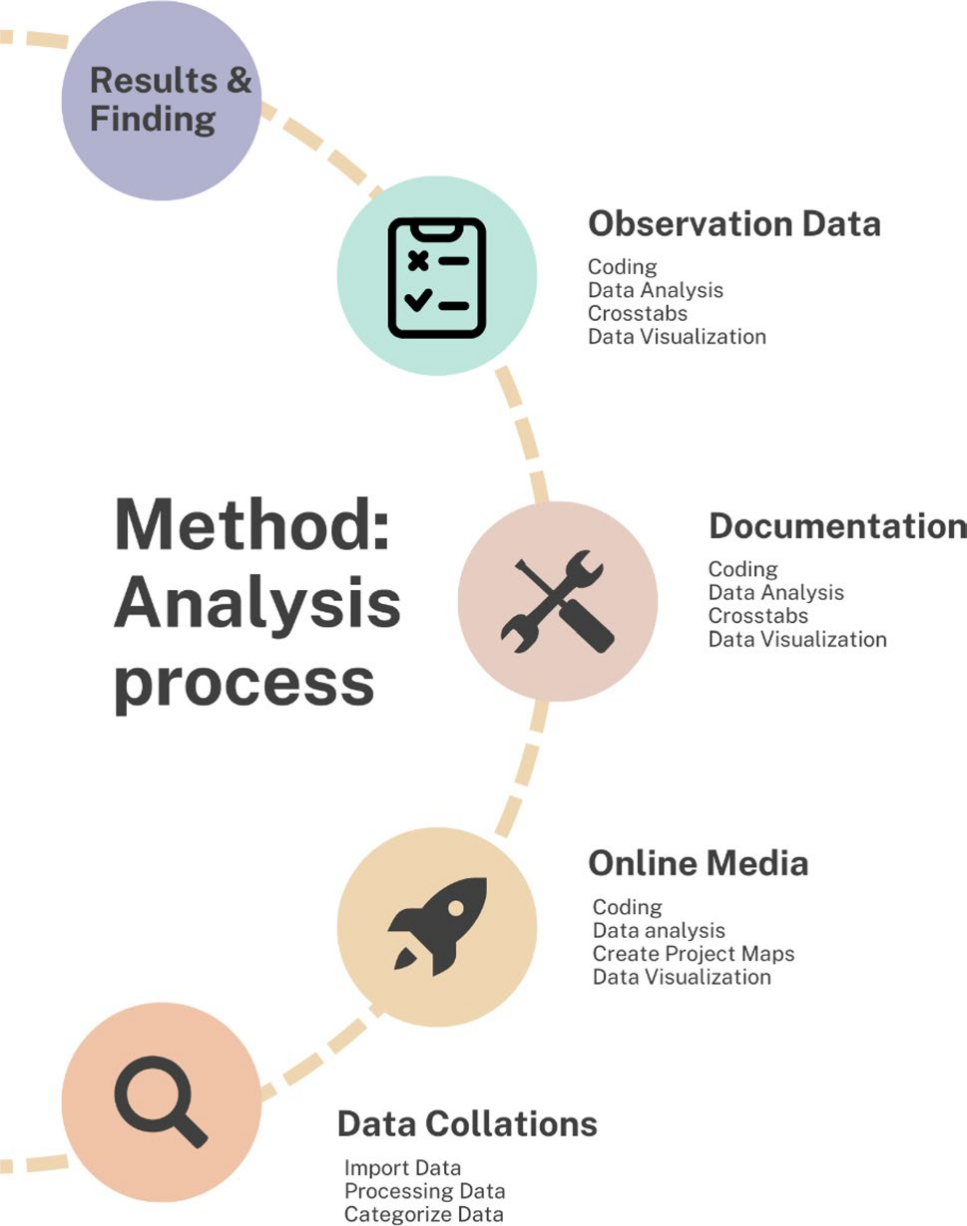
Technical Analysis Data via NVivo 12 plus. Source: processed by researchers, 2023.

Analysis using Nvivo 12 Pro was carried out in the following stages (Fig. 1): (1) data collection, (a) data import, (b) data processing, and (c) data categorization. Then (2) Media online was carried out with the following steps: (a) data coding, (b) data analysis, (c) creation of a project map, (d) data visualization, and (3) data observation & documentation with stages (a ) data coding, (b) data analysis, (c) crosstaps, and (d) data visualization. This analysis model is a qualitative analysis model using the Nvivo 12 pro tool which helps map and identify various sources of research data so that it can provide the best findings.

## Research site

**Figure 2:**
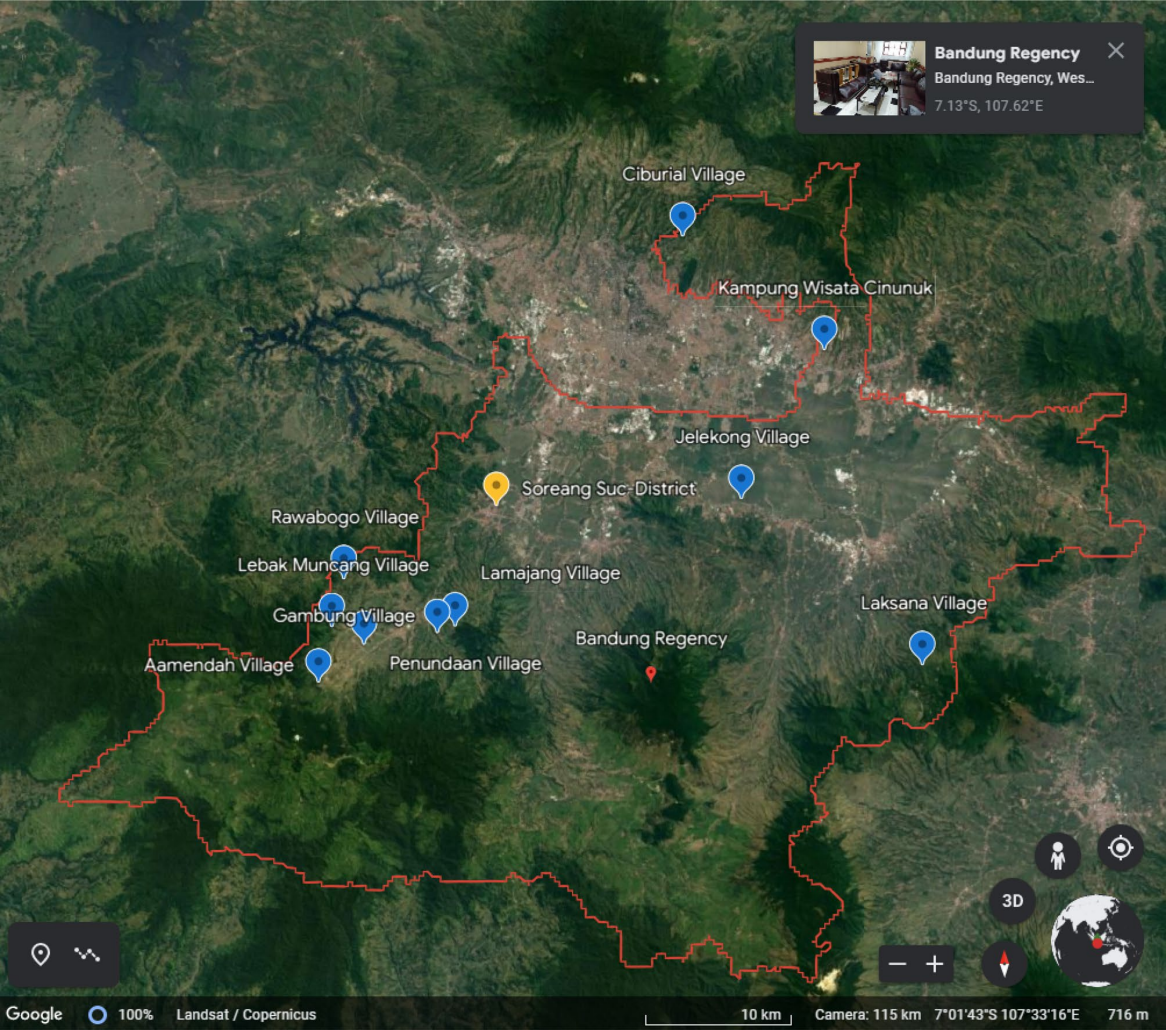
Geographical Location and 10 Tourism Villages in Bandung Regency, Indonesia. Source: Processed from google earth, 2023.

The location in this study is Bandung Regency, a district located in West Java Province, Indonesia. This location was chosen because Bandung Regency has 10 tourist villages which are pilots in various regions in Indonesia, then there is the development of 100 tourist villages, which has currently established 50 pilot tourist villages that carry the concept of sustainable tourism in Bandung Regency, Indonesia. Bandung Regency is the "mother" of the Greater Bandung area which was later divided into the City of Bandung, the City of Cimahi, and West Bandung Regency. The area is dominated by cool mountainous areas, making the natural tourist attractions in Bandung Regency very diverse. Bandung Regency is also the location of the Upper Citarum River^25^. With this geographical potential, Bandung Regency has launched a program to develop 100 tourist villages, referring to the Decree of the Regent of Bandung Regency Number 556.42/Kop7-Dispopar/2011, it has determined 10 tourism villages in Bandung Regency including Alam Endah Village, Gambung Village, Penundaan Village, Lebak Muncang Village. , Lamajang Village, Jelekong, Ciburial Village, Cibolerang Village, Laksana Village, and Rawabogo Village. This program is an effort to exploit natural and cultural potential, community empowerment, and accelerate economic growth in Bandung Regency, Indonesia. An overview of Bandung Regency, Indonesia is shown in Fig. 2:

## Results

### Empirical conditions of tourism village creative development in Bandung Regency, Indonesia

The Bandung City, not only has thousands of natural and culinary charms as well as fashion. The tourist village in Bandung Regency is also a separate attraction that must be known. Just like Yogyakarta and Bali, the potential resources in Bandung Regency are considered very feasible to be developed to become a tourist village. Not only rich with natural tourist charm, but tourist villages in Bandung Regency are also very rich in agriculture, plantations, handicrafts, painting, etc. Bandung also has several art villages and strawberry-processed foods, sprawling tea gardens, and other potential villages^26–28^.

In 2019, there were around ten tourist villages that began to be developed and designated as tourist villages by the Bandung Regency government according to the Bandung Regency Regent's Decree Number 556.42/Kop7-Dispopar/2011. The ten tourist villages in Bandung are as follows (see Table 1):

**Table 1:** Designated tourism village in Bandung Regency, West Java, Indonesia.

No	Village name	Kind of village tourism
1	Alamendah village	Agrotourism, Traditional Arts, Punceling Pass, Handicraft Processing Home Industry, Caruh Coffee (Café), Milking Cows, and Camping Grounds^29^
2	Mekarsari/Kampung Gambung village	Various strawberry processed foods, animal husbandry, handicrafts, cultural arts, fisheries, and agriculture^30^
3	Panundaan village	Rabbit farming, fisheries, agriculture, and handicrafts^31^
4	Lebakmuncang village	Education on Agrotourism, nature tourism, and Handicrafts^32^
5	Lamajang village	Traditional arts, culture, agriculture and plantations, homestays, out bond, and livestock attractions^33^
6	Jelekong village	Painting, wayang golek, and traditional culinary arts^34^
7	Ciburial village	Cultural arts, nature tourism, culinary tourism, honey bee swarms, and livestock villages (cattle farms)^13,28^
8	Cinunuk village	Arts and culinary villages^28,35^
9	Laksana village	Natural potential in the form of Kamojang Crater and agricultural and plantation products^28^
10	Rawabogo village	Natural Potential and Sites of Mount Nagara Padang and Cultural Arts^26^

Source: Based on researchers from various sources, 2023.

**Figure 3:**
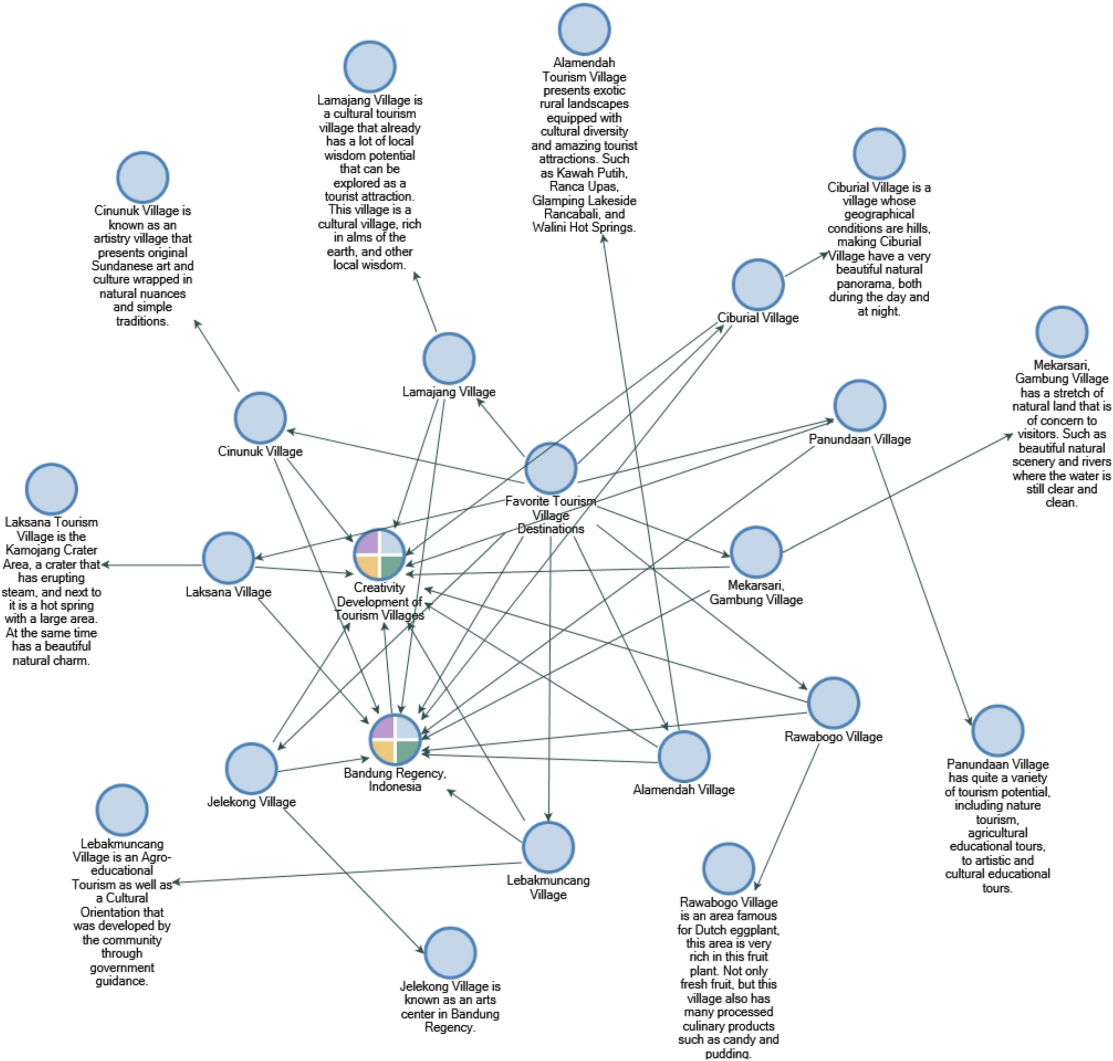
The actual condition of a creative Tourism Village in Bandung Regency, Indonesia. Source: Processed through Nvivo 12 Plus, 2023.

Not only that, but several unique tourist villages in Bandung Regency offer interesting things to explore. In Fig. 3 we will present a complete description of the 10 tourist villages in Bandung Regency, Indonesia^26–28^.

It was the conditions of the 10 villages that encouraged Bandung Regency as a tourist village in the province of West Java, Indonesia^27,28,36^. This is to maintain sustainable economic development in remote/rural areas involving various actors and development sectors in Bandung Regency, Indonesia. Development of village tourism in the development of creative villages, given its impact on economic growth and people's welfare in Bandung Regency as one of the areas with various tourist destinations^26,27^. However, tourist villages in the city have not fully received tourism attention. Therefore, solutions must be found to optimize tourism management and significantly increase the number of tourist visits, sustainability, and urban resilience in Bandung Regency, Indonesia.

### Creative development in pentahelix and quintuple helix on village tourism innovation in Bandung Regency, Indonesia

In creative development, synergy is needed between actors and the development sector^15,37^. This is by what is explained in the Penta helix concept in viewing creative and innovative development processes^15,16,37^. The Penta helix concept describes the development of collaboration between Academics, Businesses, Communities, Government, and Media to create ecosystems based on creativity and knowledge^15,16,37^. This concept is a solution for the development of creativity, innovation, and technology in the creative industries. Encouraging urban sustainability and resilience through the development of village tourism in Bandung Regency also requires adequate village environmental potential support, as meant by Carayannis and Campbell^10^. in the Quintuple Helix concept sees the environment as a driver of sustainable development innovation, therefore various efforts are being made by Bandung Regency through collaboration and synergy between creative multi-stakeholder development.

The Regent of Bandung Regency is optimistic that with the pattern of togetherness and cohesiveness between the village community and the local government, the plan to develop a tourism village will be quickly implemented^25,27^. The Bandung district government is also pushing for several locations, including adequate infrastructure in various existing tourist villages, which are mapped by the Bandung Regency DPUTR (Public Works and Spatial Planning) in each sub-district, tourist areas^25,27^. The Regent of Bandung^25,27^ said that after conducting the mapping, his party would discuss with the Bandung Regency Tourism and Culture Office and the village heads about what Bandung Regency Government should support^25,27^. Note that this tourist village can be formed when there is a desire from the bottom line and of course, it has to look at several potentials. So that it will be more inclined to maximize existing tourist attractions and their opportunities, for example in Cipelah^25,27^. Apart from that, there is also an infrastructure program to improve the quality of road construction connected to Cianjur which was launched by the Regent of Bandung Regency^25,27^.

Furthermore, the Bandung Regent has also asked several institutions such as PT. Perkebunan Nusantara (PTPN) VIII and PT. Perhutani as other parties in the creative development process of tourism villages in Bandung Regency. They play a role in widening road access for several areas which are road tracks that are under the management of PTPN and Perhutani and the involvement of investors in their development^25,27^. The government is pushing to bring in investors in the development of creative tourism villages in Bandung Regency^25,27^. It is intended that later after there are investors, such as one of them is Nimo Highland Pangalengan, a tourist spot that is busy with tourists. This has the effect of increasing employment and also extraordinary income for the community and villages^25,27^. Currently, village tourism in Bandung Regency has two artificial tourist sites, the first is Nimo Highland Pangalengan, and the second is the Rengganis Rancabali Bridge, the longest bridge in Southeast Asia^25,27,38^.

Based on these discussions referring to the Penta helix model and the Quintuple Helix concept as a driver of sustainable innovation development through tourism villages in Bandung Regency, Indonesia the role of multi actors and multi sectors is shown in Table 2.

**Table 2:** Multi-stakeholders’ role based on the quintuple helix model.

Mentor Source	Role	Objectives	Competence	Output
Creative community	Village tourism development	Increase village innovation and creativity	Understand the concept of target marketing and segmentation Able to develop new products based on the needs of tourists	Increase market share Able to carry out marketing and e-marketing capabilities (utilizing technology) Able to innovate and release new products
Local government agency	Policy and Legal Support	Engineering Training Improving the ability of the community and SMEs in managing the business Facilitator in involving other actors in the management of tourist villages	Guidance and training to the community, increase the resilience of local businesses Able to implement best practices in human resource management	Improving the ability of a better society
Social media community	Social media and information technology	Expanding small business networks and promoting tourism villages on a wider scale	Understand the advantages of joining business communities on various digital platforms	Become a member of a network of small businesses at national and international levels
Academics	Financial management and intellectual property rights	Strengthen the management structure of tourism village products Financial access to formal institutions Increase the knowledge and perception of the community in the management of tourist villages	Know and understand the phases of business institutional management and management of tourist villages	Encouraging village tourism organizations to have the following: Organizational structure Standard operational procedures Business plan Village Community Development Reliable financial reports
Business actors	Internasionalisasi pasar dan motivasi kewirausahaan	International market penetration	Attract international tourists	Understanding and working on tourism village management Become an investor in the development of a tourist village
Environment	Natural resources	Utilization of natural resources	Foundation and Support for Resilience and Sustainability	Natural capital is a foundation for carrying out development and empowerment

Source: Processed from various sources, 2023.

Based on research identification and analysis through various data sources available (as shown in Table 2), a very significant role in the development of creative tourism villages in Bandung Regency, Indonesia is played by the local community Creative and assistance & support from the local government of Bandung Regency, Indonesia. The Social Media Community also plays an important role in promoting various testament villages in Bandung Regency in attracting visitors and investors from business actors. Almost all tourist villages in Bandung Regency involve business actors in increasing the development of natural and artificial tourist villages in Bandung Regency. Another important sector, namely nature/the environment, is an important supporter of tourist villages in Bandung Regency because almost all tourist villages in Bandung Regency utilize natural tourism.

The designation of tourist villages in Bandung Regency is intended not only to improve the community's economy and village development but also to encourage the promotion of local wisdom and tourism sustainability. So village development is carried out not from top to bottom but from community participation or wishes^27,38^. These results are in line with what Hall and Williams^8^ said that breakthroughs in tourism innovation development are very dependent on the social approach taken, the importance of policy and the role of academics in it. The idea of an innovative tourist village in Bandung Regency also involves the participation of the local community, such as when the community's desire is getting higher and stronger, the local government supports and facilitates it^7,27,38^. This is confirmed in the research of Muhyi et al.^17^, Sumarto et al.^18^ that community participation and government support are the sectors with the most significant role in developing tourist villages in Bandung Regency, Indonesia. Villages that already have natural potential will be developed into better destinations while still prioritizing local village wisdom so that they can create sustainability and resilience through the tourism village program^27,38,39^. According to Verdiana^26^ explains that the tourist village development program in Bandung Regency makes more use of nature as a tourist destination and in its management prioritizes existing local wisdom. Meanwhile, villages that do not have natural potential will be developed into artificial tourism villages while still encouraging existing local cultural wisdom^27,30^ as well as developing culinary, home-made crafts, and painting in village tour packages in Bandung Regency, Indonesia. This is in line with research conducted by Purnomo et al.^37^ that tourism village developers in Indonesia, including in Bandung Regency, prioritize sustainability and management that is resilient to all kinds of threats and risks in the future.

## Discussions

### The impact of the creative development of village tourism innovation in Bandung Regency, Indonesia

In principle, the Penta-Helix model and the Quintuple Helix model add elements of government, the tourism industry, universities, media, society, and the environment that interact with each other in the management of tourist villages in Bandung Regency, Indonesia^10,18,37^. So that it becomes more optimal and attracts more tourists to visit. Collaboration and synergy between multi-stakeholders in the creative development of rural tourism in Bandung Regency create sustainability and resilience that takes into account the economic, social, infrastructure, and environmental dimensions^40,41^.

Research by Purawinata and Indratno^42^. states that success in creative development collaboration in Bandung Regency requires a clear division of responsibilities for each development actor and encourages good communication to encourage maximum participation. Nasution et al.^43^ stated that the development of tourist villages in Indonesia requires the resilience of social networks that really support increasing institutional capacity (Village Government, Local Institutions, and development practitioners), fostering collaboration, and requiring creativity. Kesa^44^. states that the process of interaction of various actors in tourism development in Bandung Regency is still not optimal, as academic roles are still lacking in the process of creative tourism development. Although actors such as local government, local communities, and the private sector have played a significant role in development. So that these findings become notes and insights into increasing the creative development of tourist villages in Bandung Regency. According to Sumarto et al.^18^ stated that the majority of tourism trends in Indonesia promote arts and culture as tourism icons. This is also not far from Bandung Regency, but several tourist villages in Bandung Regency also promote nature tourism, animal husbandry, and agricultural education, such as Laksana Village in Kec. Ibun, Rawabogo Village in Kec. Ciwidey, Ciburial Village in Kec. Cimenyan, and Lebakmuncang Village in Kec. Ciwidey.

**Figure 4:**
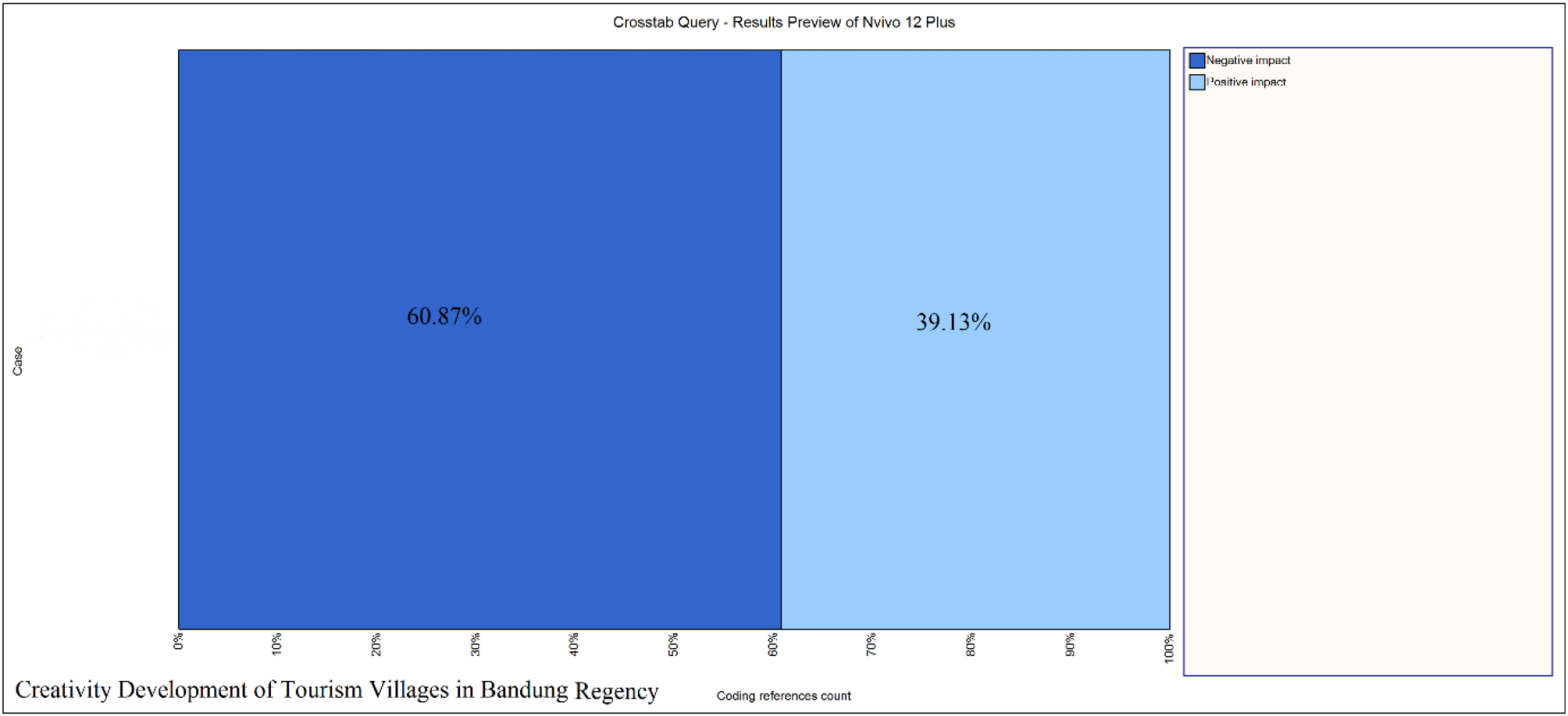
Percentage of the Impact of Creative Tourism Village Development in Bandung Regency. Source: Processed through Nvivo 12 Plus, 2023.

In the business of implementing and developing tourism, important elements of tourism must carry out their respective functions by carrying out maximum planning and implementation. The development of the village tourism potential in Bandung Regency certainly has an impact on residents, both positive and negative impacts^42,43,45–47^. As follows:

Based on the results of the identification and analysis in Fig. 4 which was processed from various data sources owned by researchers, it shows that the percentage of negative impacts is still higher than the positive impacts in creative development and management of tourism villages in Bandung Regency. This shows that better management is still needed in the tourist villages in Bandung Regency, Indonesia. Some of the negative impacts that need attention are (1) Environmental impacts that occur; (2) Villagers are not the owners of tourist sites because there is a lot of interference from outsiders, residents are only workers in several tourist villages; (3) The number of tourism that has not increased optimally in several tourist villages; (4) people's perception and knowledge of innovation and creativity in the management of tourism villages is not good enough; (5) Limited support for facilities and infrastructure; (6) Several village potentials that have not been utilized; (7) Mismanagement in the management of tourist villages which resulted in a decrease in the number of tourists in several tourist villages; (8) Utilization of Technology that has not been maximized. Even so, the creative development of tourist villages in Bandung Regency has positive impacts, such as (1) creating jobs for local communities; (2) improving the economy; (3) introducing local products and culture; (4) preserving local culture; (5) central government support; (6) Involve various development actors. Based on the results of the identification and analysis of this research, it can be said that the development of creative tourism villages in Bandung Regency must of course be planned and prepared well with all the impacts that will arise. This is so that the development and management of village potential can benefit everyone, and the goals of developing tourist villages can be realized.

Indonesia is currently focused on developing tourism to increase economic growth and community welfare through various tourism village programs, one of which is in Bandung Regency. This is due to the good tourism potential of Bandung Regency to become a tourist village^27^. The majority of tourist destinations in Bandung Regency promote art and culture as tourism icons, and several tourist villages in Bandung Regency also promote nature tourism, animal husbandry, and agricultural education, such as Laksana Village in Kec. Ibun, Rawabogo Village in Kec. Ciwidey, Ciburial Village in Kec. Cimenyan, and Lebakmuncang Village in Kec. Ciwidey needs good management by paying attention to the principles of sustainability and resilience. The current management of tourist villages is not yet optimal but shows a positive side such as the collaboration of various multi-stakeholders between the government, industry, community, media, universities, and the environment. This is in line with research conducted by Sumarto et al.^20^ and Abdillah et al.^7^ with this collaboration, the management of tourist villages can be more optimal and able to attract tourists to visit which has an impact on urban sustainability and resilience as long as it pays attention to strengthening various dimensions such as social, economic, infrastructure and environmental dimensions in Bandung Regency, Indonesia.

### Implications of practice, theoretical and institutional

The practical implications of this research show that the creative development program for tourist villages in Bandung Regency, Indonesia is directed at realizing sustainability (social, economic, and environmental) and resilience (against all threats and disturbances in the future). The creative development program in Bandung Regency, Indonesia is the result of various interactions and relationships that are consistently carried out by various sectors such as local communities, government, online media communities, business actors, and the environment, although in the process there are still weaknesses such as the involvement of each sector which is still uneven. impact on development programs not being optimal. The theoretical implication that we underline is in line with the quintuple helix concept that nature/the environment needs to be aligned with other important sectors in realizing sustainable and resilient development interactions. The institutional implications of this research underline that creative development programs in Bandung Regency, Indonesia can only be carried out well if they are carried out with clear coordination and job descriptions for the institutions and agencies involved, so that there will be no overlap in duties and responsibilities.

## Conclusion

From the analysis of the tourism sector that has been carried out in various tourist villages in Bandung Regency, it can be concluded that in developing tourism potential in an area, of course, one must think carefully about the impact on the surrounding community, so that the development of tourism potential does not only benefit one party but the benefits can be felt. by the local community equally. The government still needs to be constrained in coordination and communication to maximize creative development in 10 tourist villages in building village tourism potential. The guard village government needs to take part in mentoring and training the community as well as building deliberations with the community in planning solutions for any negative impacts that may occur.

Based on this analysis and support, the management of Bandung Regency as a tourism village requires the synergistic collaboration of several elements including the government, the tourism industry, universities, media, community, and the environment which promotes sustainability and resilience which needs to pay attention to strengthening the social, economic, infrastructure dimensions. , and the environment. This interaction can at least be a solution to improving the quality of tourism village management in Bandung Regency, Indonesia. With this, the government of the tourism industry, universities, media, society, and the environment that interacts synergistically with each other in the management of tourist villages in Bandung Regency will attract more tourists to visit, a sustainable environment, and increase regional resilience from disturbances such as natural disasters, local economic problems, and future unemployment. We recommend that in order to overcome the various impacts that occur in the development and management of tourist villages in Bandung Regency, strong commitment and support from various parties is needed in minimizing the negative impacts that occur. In this way, the solution to any negative and positive impacts that occur can be managed well and appropriately.

This research is limited to field data such as in-depth interviews with development actors from various parties such as the government, the tourism industry, universities, the media, and community leaders in the process of managing and developing tourist villages in Bandung Regency, Indonesia.

## Data Availability

The results of the study can be found in the figures attached to the article. The data set used to support the findings of this study is available from the corresponding author upon request.
